# RNA-seq-based transcriptome profiling of early fruit development in Chieh-qua and analysis of related transcription factors

**DOI:** 10.1038/s41598-024-63871-6

**Published:** 2024-06-12

**Authors:** Xuan Du, Na Liu, Panling Lu, Ying Wang, Bo Lu, Shoubo Tian, Zhaohui Zhang

**Affiliations:** 1 Horticultural Research Institute, Shanghai Academy of Agricultural Sciences, Shanghai Key Laboratory of Protected Horticultural Technology, Shanghai, 201403 China; 2 Information Research Institute of Science and Technology, Shanghai Academy of Agricultural Sciences, Shanghai, China; 3 Zhuanghang Comprehensive Experiment Station, Shanghai Academy of Agricultural Sciences, Shanghai, China

**Keywords:** Transcriptome, Early fruit development, Chieh-qua, Transcription factors, MYB_superfamily, Plant sciences, Plant development, Plant morphogenesis

## Abstract

Chieh-qua (*Benincasa hispida* Cogn. var. Chieh-qua How.) fruit development starts post pollination. With the continuous expansion of the fruit, the soluble solid content of the fruit decreases. Because there are no reports on the early development of Chieh-qua fruit, this study compared fruit transcriptomes at 0-, 3-, and 7 day post pollination (dpp). 104,747 unigenes were assembled from clean reads and compared using six public databases for similarity searching. Compared with those of 0 dpp (C), there were differences in the expression of 12,982 and 6541 genes in the fruit tissue at 3 dpp and 7 dpp, respectively. Compared with 3 dpp (B), there were 14,314 differentially expressed genes in the fruit at 7 dpp (A). Based on the analysis of transcription factors, 213 nucleotides in the MYB superfamily were identified; among them, 94 unigenes of the MYB superfamily were differentially expressed at the three stages. In the pairwise comparison of differential expression, eight unigenes (Gene_id: TRINITY_DN32880_c1_g2, TRINITY_DN35142_c2_g2, TRINITY_DN32454_c11_g6, TRINITY_DN34105_c2_g7, TRINITY_DN32758_c3_g3, TRINITY_DN33604_c4_g10, TRINITY_DN34466_c3_g1, TRINITY_DN35924_c3_g2) were homologous to those of MYB59, MYB-GT3b, MYB18, MYB4, MYB108, MYB306, MYB340, and MYB-bHLH13. These unigenes differed significantly among the three stages. Furthermore, MYB59 and MYB18 exhibited higher expression at 7 dpp. MYB4, MYB-GT3b, MYB108, and MYB306 showed the highest expression levels in fruits at 3 dpp. In addition, MYB340 and MYB-bHLH13 showed higher expression levels during the unpollinated stage. MYB59, MYB-GT3b, MYB18, MYB4, MYB108, MYB306, MYB340, and MYB-bHLH13 may play crucial roles in Chieh-qua fruit development, defense, and blossoming. This study provides a basis for further investigation of MYB superfamily genes involved in early fruit expansion in chieh-qua.

## Introduction

Chieh-qua (*Benincasa hispida* Cogn. var. Chieh-qua How.), a vigorous annual vine with dioecious flowers, is a subspecies of wax gourd (*Benincasa hispida* (Thunb.) Cogn.)^1^. This vegetable crop belongs to the *Cucurbitaceae* family and is widely distributed in South China and Southeast Asia^2,3^. The immature or mature fruit is consumed and can be boiled, fried, or pickled. Chieh-qua is also a traditional Chinese medicine in the Materia Medica compendium. Modern research has shown that its juices have hypotensive effects^4^.

Fruit development begins after pollination and fertilization^5^. Fruit development is generally divided into two stages, early fruit development and fruit ripening, which include changes in fruit size, shape, nutritional components, and texture. This study focused on the expansion and development of early fruit, including (i) fruit setting, (ii) fruit growth through cell division, and (iii) fruit growth through cell expansion^6,7^. Rapid cell division and elongation occur during the growth stage and determine the final size and shape of the fruit^8^. Fruit size and shape are among the most important characteristics determining fruit market value^7^. The shape and size of fruits are regulated by molecular mechanisms that regulate fruit development.

During fruit development, the general fruit size, shape, and other changes depending on organ characteristics are determined at an early stage. Various transcription factors regulate this process. In the process of tomato fruit development, MYB factor FSM1 participated in the negative regulation of fruit size in the early development of tomato fruit^9^. In addition, the transcription factors NON-RIPENING (NOR), COLORLESS NON-RIPENING (CNR), and RIPENING INHIBITOR (MADS-RIN) play regulatory roles in tomato fruit ripening^6^. Therefore, transcription factors may play important roles during the early stages of fruit development.

In recent years, transcriptome sequencing has been applied to fruit development in Cucurbitaceae crops, such as cucumber, melon, and bottle gourds, to study the molecular transcriptional regulation of fleshy fruit development^7,10–12^. However, most studies have focused on fruit ripening, size, and flavor, and few studies on the early development of fruits have studied tomatoes and cucumbers, not chieh-que^10,13^.

In this study, we studied the differences in transcription levels between two fruit sizes. The purpose was to reveal transcriptional changes during the early stages of Chieh-qua fruit development. In our opinion, development is a dynamic process and a differential change before and after an individual’s development. Therefore, we focused on the differences in transcription levels before and after pollination during the early stages of fruit development. We also attempted to identify the regulatory factors related to the early expansion of Chieh-qua fruit. We expect the information generated in this study to facilitate the development of gourd breeding programs to improve fruit size.

## Material and methods

### Chieh-qua plant and its fruits’ early development measurements

The chieh-quas “Verdant No. 1” used in this research were originally obtained from Zhongziku APP (http://www.zhongziku.cc/) commercially. Chieh-qua seeds were sown on March 10 and planted at the Zhuanghang Experimental Station of Shanghai Academy of Agricultural Sciences on April 18. The photon flux density was range 650 ~ 850 W.m^−2^. The temperature and relative humidity of the cultivation environment was about 10 ~ 25 °C and 50 ~ 70%, respectively. Fruits were selected at 0-, 3-, and 7 day post pollination (dpp), and the weight of the fruit was measured with a scale. The pericarp of fruits was removed, then the pulp was homogenized, and the soluble solid content of the fruits was measured with a handheld sugar meter (Master-a, Japan).Subsequently, the fruits were harvested and washed under running tap water followed by sterile water, sampled with a knife, wrapped in foil paper, put into liquid nitrogen, and stored in a refrigerator at – 86 ℃ for subsequent analysis^14^. Each sample had three biological replicates and each replicate included five individual plants.

### RNA extraction, library construction, and sequencing

RNA was extracted using the RNAprep Pure Polysaccharide Plant Total RNA Extraction Kit (DP441; Tiangen Biotechnology, Beijing, China), following the manufacturer’s protocol. The quantity and quality of RNA were determined using a NanoPhotometer^®^ NP80 (IMPLEN, CA, USA). The RNA integrity number (RIN) was measured by an Agilent 2100 Bioanalyzer (Agilent Technologies, Palo Alto, CA, USA). All extracted RNA samples reached the total amount ≥ 1 ug, the concentration ≥ 35 ng/μL, OD260/280 ≥ 1.8, and OD260/230 ≥ 1.0.

First, mRNA was separated from total RNA by A-T base pairing the magnetic beads with oligo (dT) and poly-A. Next, fragmentation buffer was added to randomly fragment the mRNA, and a small fragment of approximately 300 bp was separated using magnetic bead screening. Under the action of reverse transcriptase, six-base random primers were added, and mRNA was used as a template to reverse synthesize one-strand cDNA, followed by two-strand synthesis to form a stable double-stranded structure. End Repair Mix was added to make the sticky end of double-stranded cDNA into a flat end; next, an “A” base was added to the 3’ end to connect the Y-shaped linker. Polymerase chain reaction (PCR) and agarose gel separation enriched and recovered the target fragments. Target fragments were quantified using TBS380 (Picogreen) and mixed according to the data ratio by using a computer. Bridge PCR amplification was conducted on cBot to generate clusters, which were subjected to Illumina NovaSeq 6000 sequencing^15^. The Illumina platform converts the sequenced image signals into text signals by using CASAVA Base Calling and stores them in the FASTQ format as raw data.

### Sequence reads de novo assembly, raw data statistics, and quality control

The software fastx_toolkit_0.0.14 (http://hannonlab.cshl.edu/fastx_ toolkit/) was used to count the base distribution and quality fluctuation in each cycle of all sequencing reads, which can visually reflect the library construction quality and sequencing quality of sequencing samples from a macro perspective, and analyze the base quality, base error rate, and base distribution of each sample^16^.

The original sequencing data contains sequencing linker sequences, low-quality reading segments, sequences with high N (N represents uncertain base information) rates and sequences that are too short, which seriously affects the quality of subsequent analysis. Therefore, the original sequencing data were quality controlled before analysis to obtain high-quality clean data and ensure the accuracy of the subsequent analysis results. Software fastp was used to remove the connector sequence from reads, reads without inserted fragment due to connector self-connection, the base with low quality (Q-score < 20) at the end of the sequence (3’ end), reads with N ratio exceeding 10% and reads with length less than 30 bp.

All clean data were assembled using Trinity software (https://github.com/trinityrnaseq/trinityrnaseq)^17^. TransRate and CD-HIT were used to optimize and filter the obtained initial assembly sequences^18^. Benchmarking Universal Single-Copy Orthologs BUSCO (http://busco.ezlab.org) was used to evaluate the assembly integrity of the transcriptome^19^. Statistical analyses were performed on the lengths of the assembled transcripts to visually obtain the length distribution of all transcripts. The clean reads of each sample were compared with the reference sequence assembled using Trinity to obtain the mapping result for each sample, which was also the basis for the subsequent quantification of genes and transcripts of each sample.

### Assembled unigenes/transcripts annotation, expression analysis, and TF identification and expression analysis

The assembled transcriptome sequences were compared with those in six databases (NR, Swiss-Prot, Pfam, COG, GO, and KEGG). The annotation information in each database was obtained, and the annotation situation in each database was counted. Quantitative analysis of the expression level of nuigenes/transcripts was conducted using RSEM software to analyze the differential expression of nuigenes/transcripts among the samples and to reveal the regulation mechanism of genes by combining the sequence function information^20^. Differentially expressed genes (DEGs) were analyzed using DESeq2, and the P-value threshold in multiple tests was determined using the false discovery rate (FDR)^21^. Based on p-adjust < 0.05 & |log2FC|> = 1, genes/transcripts with different expression between groups were obtained by Transcripts Per Million reads (TPM)^22^.

By analyzing the domain information contained in transcripts, gene/transcripts can be predicted for transcription factors and family analysis and then compared with the database Plant TFDB 4.0 (http://planttfdb.cbi.pku.edu.cn/) by using the HMMER analysis method to obtain homologous transcription factor information^23^. The expression difference analysis method for TFs was the same as that for the aforementioned differential genes/transcripts.

### Quantitative real-time PCR validation of expression of MYB_superfamily

Total RNAs were isolated using the RNAprep Pure Polysaccharide Plant Total RNA Extraction Kit (DP441; Tiangen Biotechnology, Beijing, China), according to the manufacturer’s instructions. cDNA was synthesized from 3 μg of each total RNA by using the Hifair ^®^ II 1st Strand cDNA Synthesis Kit (Yisheng Biotechnology, Shanghai, China), according to the manufacturer’s instructions. qRT-PCR was performed using a QuantStudio 5 (ABI, USA) in combination with a UNICON ^®^ Power qPCR SYBR Master Mix (Yisheng Biotechnology, Shanghai, China). The primers used to detect Myb transcripts are listed in File S1. The qRT-PCR method was as follows: initial 95 °C enzyme activation for 30 s, followed by denaturation for 10 s at 95 °C, and annealing/extension 30 s at 60 °C for 40 cycles. Data were collected using the QuantStudio 5 Manager software and processed in Microsoft Excel. The transcript levels of target genes were normalized first to the transcript levels of the constitutively expressed *Bhactin* gene and then to *myb* gene transcripts in A (negative control) according to the 2^−ΔΔCt^ method^24^.

### Source of plants statement

The Chieh-qua varietie used in the study were complied with relevant institutional, national, and international guidelines and legislation. And the study also complies with the IUCN Policy Statement on Research Involving Species at Risk of Extinction and the Convention on the Trade in Endangered Species of Wild Fauna and Flora for collection of seed specimens used in the study.

## Results

### Chieh-qua development and sampling

**Figure 1:**
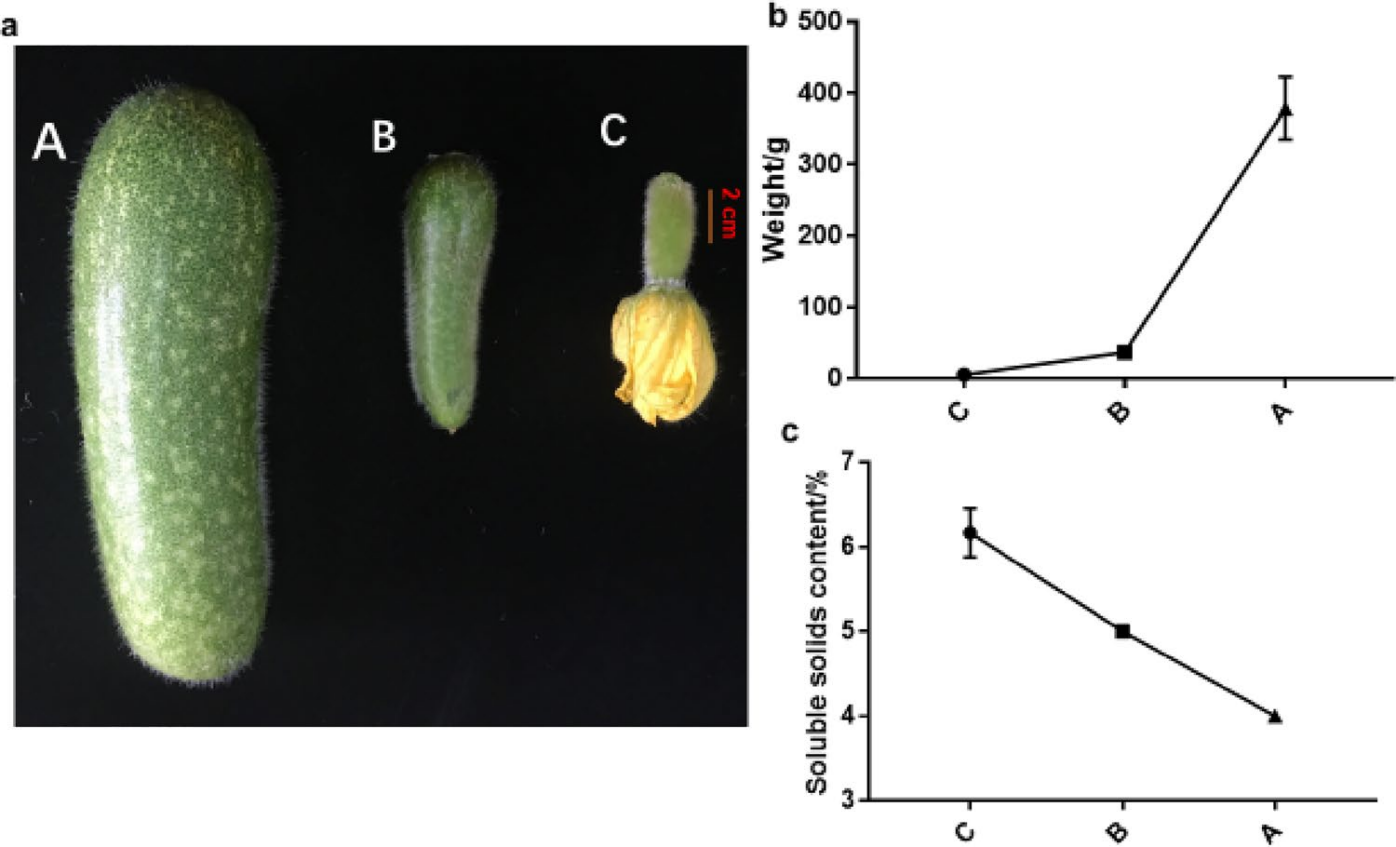
Early fruit development after pollination. Changes in appearance (**a**), weight (**b**), and soluble solids content (**c**) during Chieh-qua fruit early development, including 7 (A), 3 (B), and 0 dpp (C).

Throughout the growth season of chieh-qua, were sampled at three stages, 7 dpp (days post pollination) (A), 3 dpp (B), before pollination 0 ddp(C) (Fig. 1a). Chieh-qua development is monitored by the measurement of Chieh-qua fruit weight and soluble solids content. After pollination, the weight of the fruit increases slowly in the first three days, but it increases greatly at 3 and 7 dpp (Fig. 1b). However, the content of soluble solids decreases with the increase of fruit (Fig. 1c). Given our interest in the transcriptional changes that may be involved in regulating fruit early enlargement, we select unpollinated fruits and fruits at 3 dpp and 7dpp for RNA-seq.

### Sequencing and de novo transcriptome assembly

To obtain the transcriptome expression profile of chieh-qua fruit development, we constructed a non-normalized library using fruits at different developmental stages, from 0 to 7 dpp. Illumina sequencing data from the Chieh-qua fruits were deposited in the NCBI SRA database under the accession number PRJNA970527. In total, 536977732 Illumina PE raw reads were generated (Table 1). After removing the adaptor sequences, ambiguous nucleotides, and low-quality sequences, 533.845180 million clean reads remained. The assembly of clean reads resulted in 104,747 unigenes in the range from 201 to 14,209 bp, with an N50 length of 2119 bp (Table 2).

**Table 1:** Summary of sequences analysis.

Sample	Raw reads	Raw bases	Clean reads	Clean bases	Error rate (%)	Q20 (%)	Q30 (%)	GC content (%)
A1	57,582,732	8.69G	57,255,182	8.56G	0.0232	98.86	95.95	44.32
A2	63,237,646	9.55G	62,845,702	9.39G	0.0235	98.73	95.56	44.54
A3	57,047,340	8.61G	56,679,826	8.47G	0.0231	98.86	95.97	44.6
B1	58,379,262	8.82G	58,016,168	8.68G	0.0237	98.66	95.37	44.52
B2	58,940,310	8.9G	58,543,660	8.73G	0.0235	98.72	95.61	44.57
B3	61,404,706	9.27G	61,052,804	9.12G	0.0235	98.71	95.52	44.1
C1	63,277,140	9.55G	62,964,486	9.42G	0.0232	98.84	95.88	44.6
C2	59,538,590	8.99G	59,241,530	8.86G	0.0232	98.85	95.91	44.72
C3	57,570,006	8.69G	57,245,822	8.55G	0.0233	98.82	95.84	44.71

A1, A2, A3:7 dppfruit tissue. B1, B2, B3:3 dpp fruit tissue. C1, C2, C3:0 dpp fruit tissue. Q20: Percentage of bases with a Phred value > 20. Q30: Percentage of bases with a Phred value > 30.

**Table 2:** BLAST analysis of transcripts and unigenes against public databases.

	Transcript number(percent)	Unigene number(percent)
NR	90,618(0.5619)	43,773(0.4179)
Swiss-Prot	82,407(0.5109)	44,845(0.4281)
Pfam	67,718(0.4199)	34,580(0.3301)
COG	24,772(0.1536)	11,483(0.1096)
GO	51,119(0.317)	21,944(0.2095)
KEGG	53,955(0.3345)	31,076(0.2967)
Total_anno	104,528(0.6481)	56,807(0.5423)
Total	161,282(1)	104,747(1)

### Sequence annotation

By comparing with 6 public databases for similarity searching, 161,282 transcripts and 104,047 unigenes are obtained. Analyses show that 44,845 unigenes (41.79%) have significant matches in the Swiss-Prot database, 11,483 (10.96%) in the COG database. Our results also show that 43,773 (41.79%) of non-redundant unigenes demonstrate similarity to the known genes in NR database. In total, there are 56,807 unigenes (54.23%) successfully annotated in at least one of the NR, Swiss-Prot, Pfam, COG, GO and KEGG databases (Table 1).

**Figure 2:**
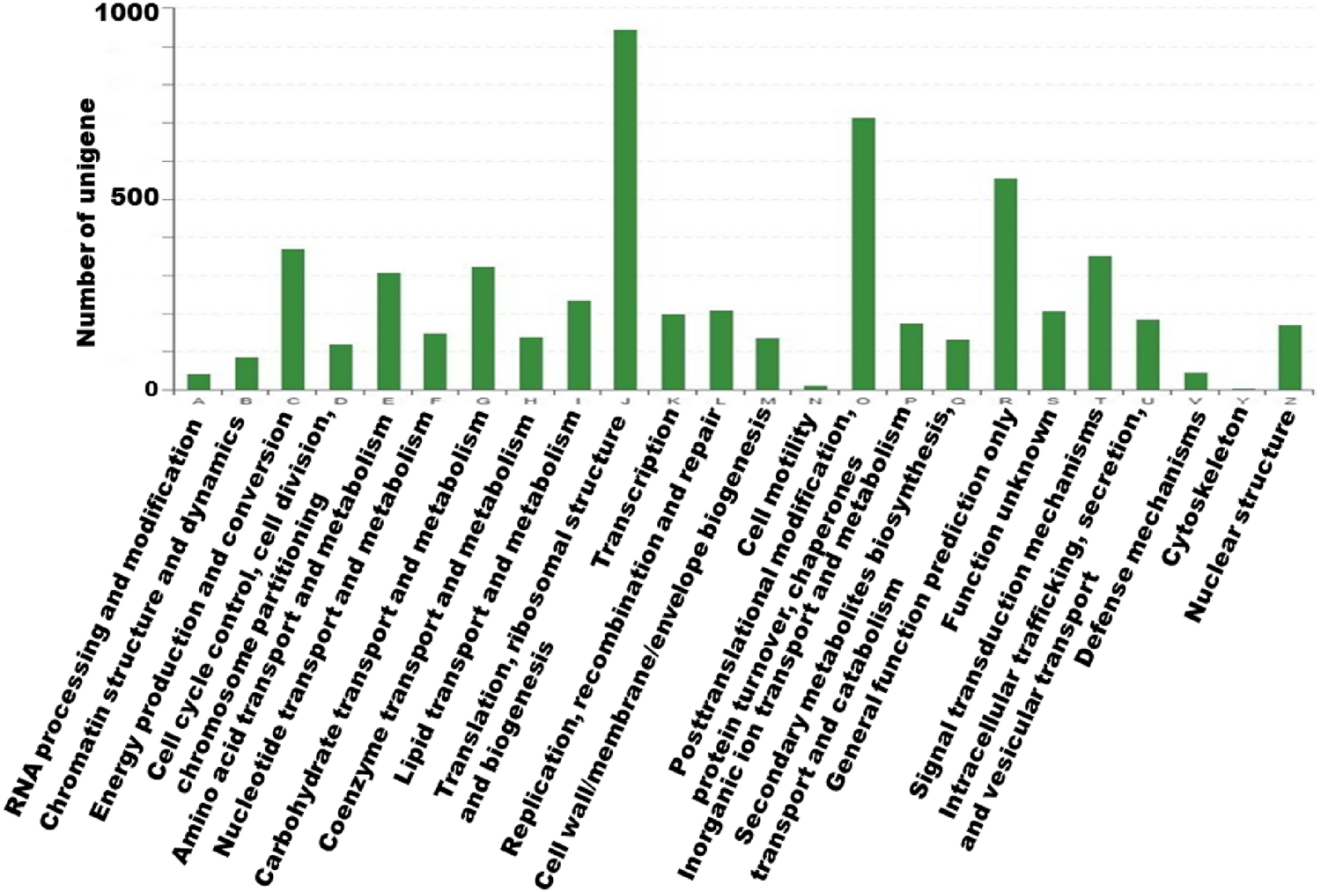
COG annotation of putative proteins.

Additionally, all the unigenes were searched against the COG database for functional prediction and classification. A total of 11,483 non-redundant unigenes (Table 2) were subdivided into 24 COG classifications (Fig. 2). Among these classifications, the cluster of ‘Translation, ribosomal structure and biogenesis’ (946) is the largest group, followed by ‘Posttranslational modification, protein turnover, chaperones’ (715), ‘General function prediction only’ (556), ‘Energy production and conversion’ (371), and ‘Signal transduction mechanisms’ (353). Only a few unigenes were assigned to cell motility (12) or nuclear structure (5).

**Figure 3:**
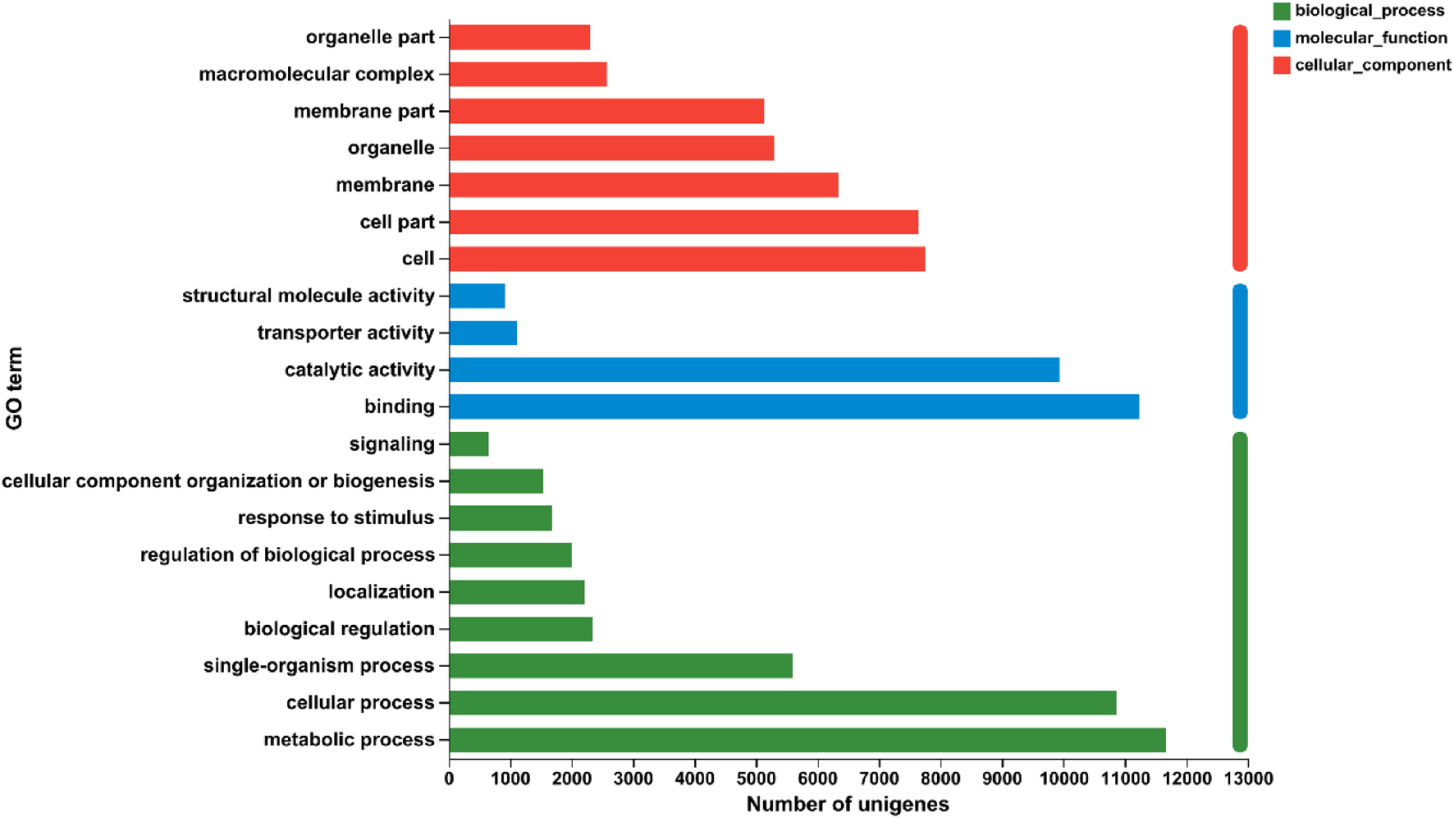
GO categorization of non-redundant unigenes. Each annotated sequence was assigned at least one GO term.

According to the International Standardized Gene Functional Classification System GO database, 21,944 non-redundant unigenes were classified into three major functional ontologies: biological processes, cellular components, and molecular functions (Fig. 3). For biological processes, the dominant subcategories were ‘metabolic process’ (11,676), ‘cellular process’ (10,866), and ‘single-organism process’ (5599). In the category of cellular component, ‘cell’ (7756), ‘cell part’ (7645), and ‘membrane’ (6345) were highly represented. Among the molecular function terms, ‘binding’ (11,234) and ‘catalytic activity’ (9934) are the most represented. However, within each of the three categories, few genes are assigned to the subcategories of ‘rhythmic process,’ ‘nucleoid’ and ‘metallochaperone activity.’

**Figure 4:**
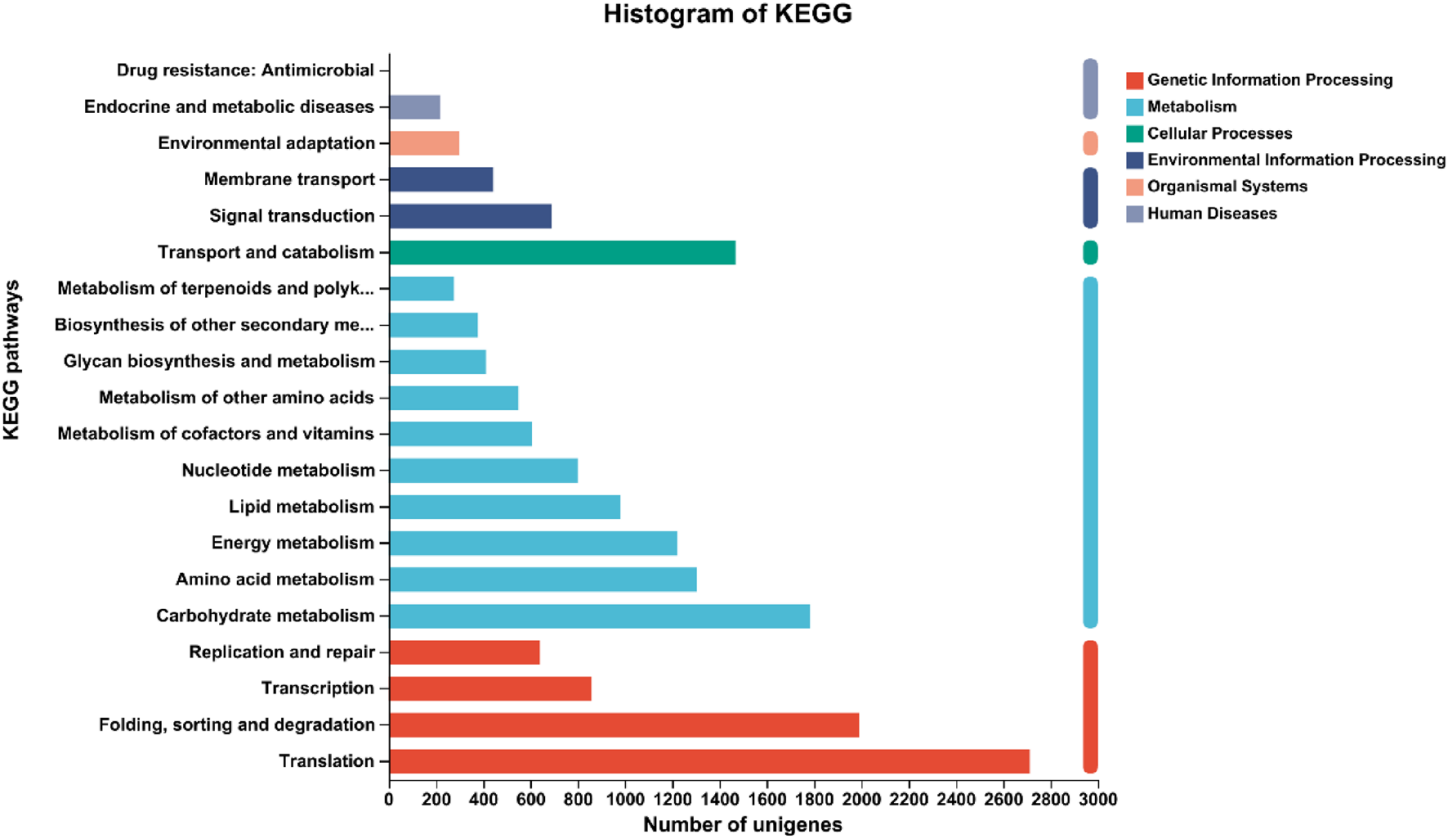
KEGG annotation of putative proteins.

KEGG is a large-scale knowledge base for systematic analysis of gene function, linkage of genomic information, and functional information. According to KEGG, 31,076 unigenes (Table 2) were assigned to six major metabolic pathways (Fig. 4). The pathways involving the largest number of unique transcripts were ‘translation’ (2712), followed by ‘folding, sorting and degradation’ (1992); ‘drug resistance: antimicrobial’ (2) was the smallest group.

### Differential expression analysis of assembled early development of Chieh-qua fruits

**Figure 5:**
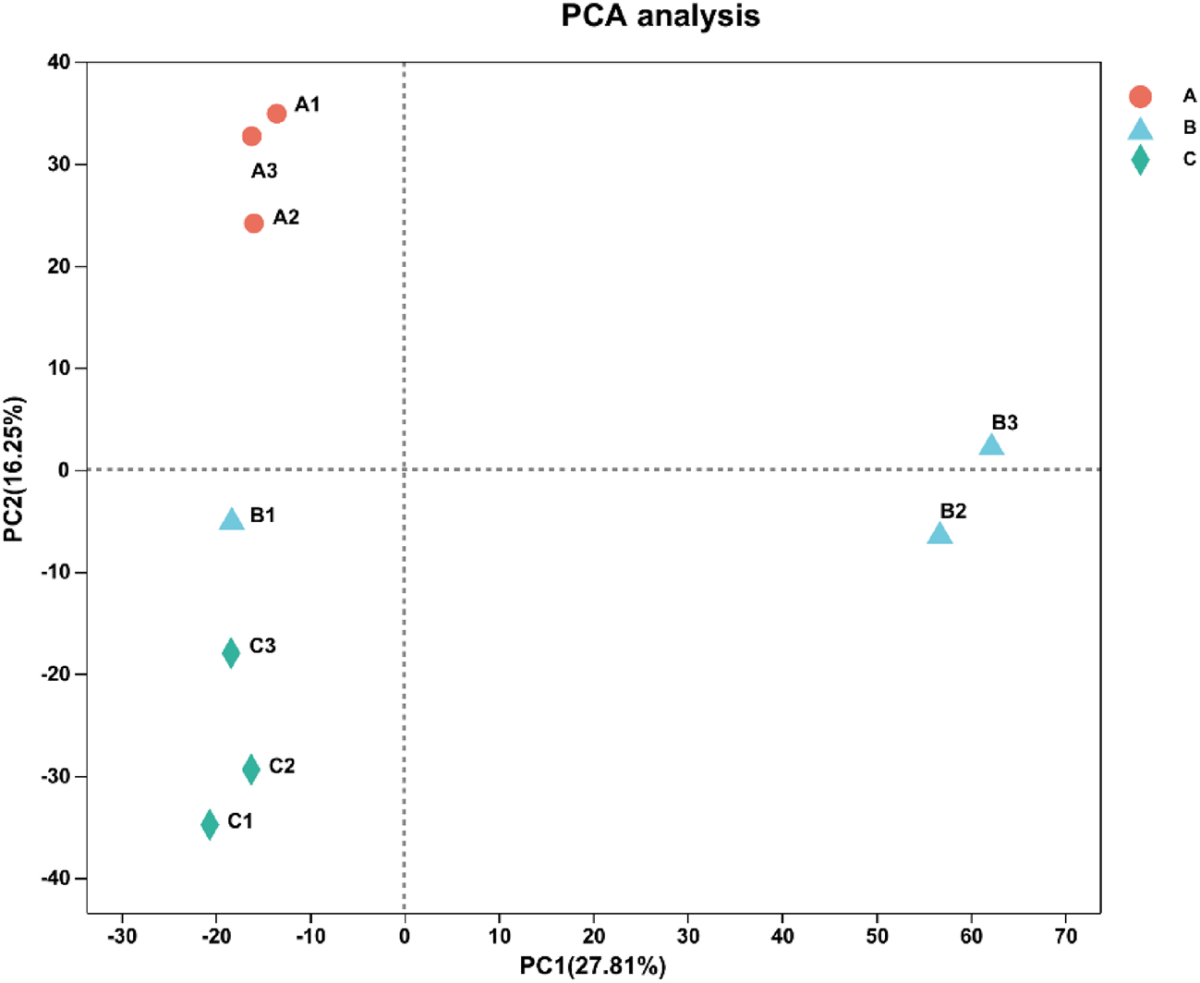
PCA analysis between samples.

To survey the biological mechanism of the early development of Chieh-qua fruits after pollination, identifying the DE genes among the three different developmental stages is necessary. To improve the accuracy of the measured expression levels for further analysis, we merged the data from three biological replicates and calculated the fragments per kilobase per million (FPKM) value based on the merged dataset. Three biological replicates of samples A and C clustered together, and two biological replicates of sample B clustered together in the PCA (Fig. 5). Based on the quantitative results of expression level, the genes with different expression between the two groups were analyzed; the difference analysis software used was DESeq2, and the screening threshold was |log2FC|> = 1 & p adjust < 0.05.

**Figure 6:**
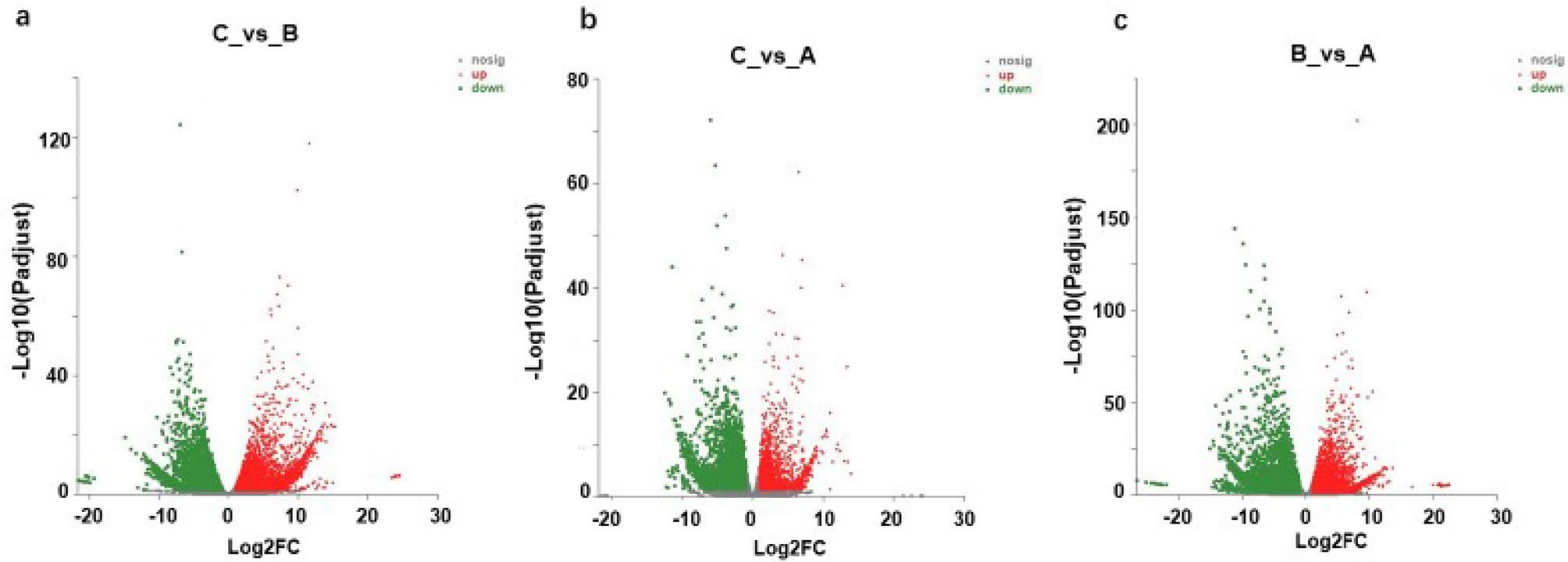
Volcano plot displaying differential expressed genes between two developing stages. The abscissa is the multiple change value of gene expression difference between two samples, that is, the value obtained by dividing the expression number of treated samples by the expression number of control samples, and the ordinate is the statistical test value of gene expression difference, that is, the P value. The higher the P value, the more significant the expression difference, and the values of horizontal and vertical coordinates are all logarithmic. Each dot in the graph represents a specific gene, the red dot represents a significantly upregulated gene, the green dot represents a significantly downregulated gene, and the black dot represents a non-significant difference gene.

Subsequently, the DEGs of the three different fruits in the early developmental stages were analyzed (Table 3, Fig. 6). There were differences in the expression of 12,982 genes in the fruit tissue at 0 (C) and 3 dpp (B), among which 6035 genes were upregulated, and 6947 genes were downregulated (Fig. 6a). Compared with 0 dpp (C), there were 6541 DEGs, of which 2479 were upregulated, and 4062 were downregulated (Fig. 6b). Compared with 3 dpp (B), there were 14,314 DEGs in the fruit at 7 dpp (A), of which 6873 genes were upregulated, and 7441 genes were downregulated (Fig. 6c).

**Table 3:** Statistics of differential gene number.

different_group	Total DEGs	up	down
C_vs_B	12,982	6035	6947
C_vs_A	6541	2479	4062
B_vs_A	14,314	6873	7441

### Analysis of transcription factors

**Figure 7:**
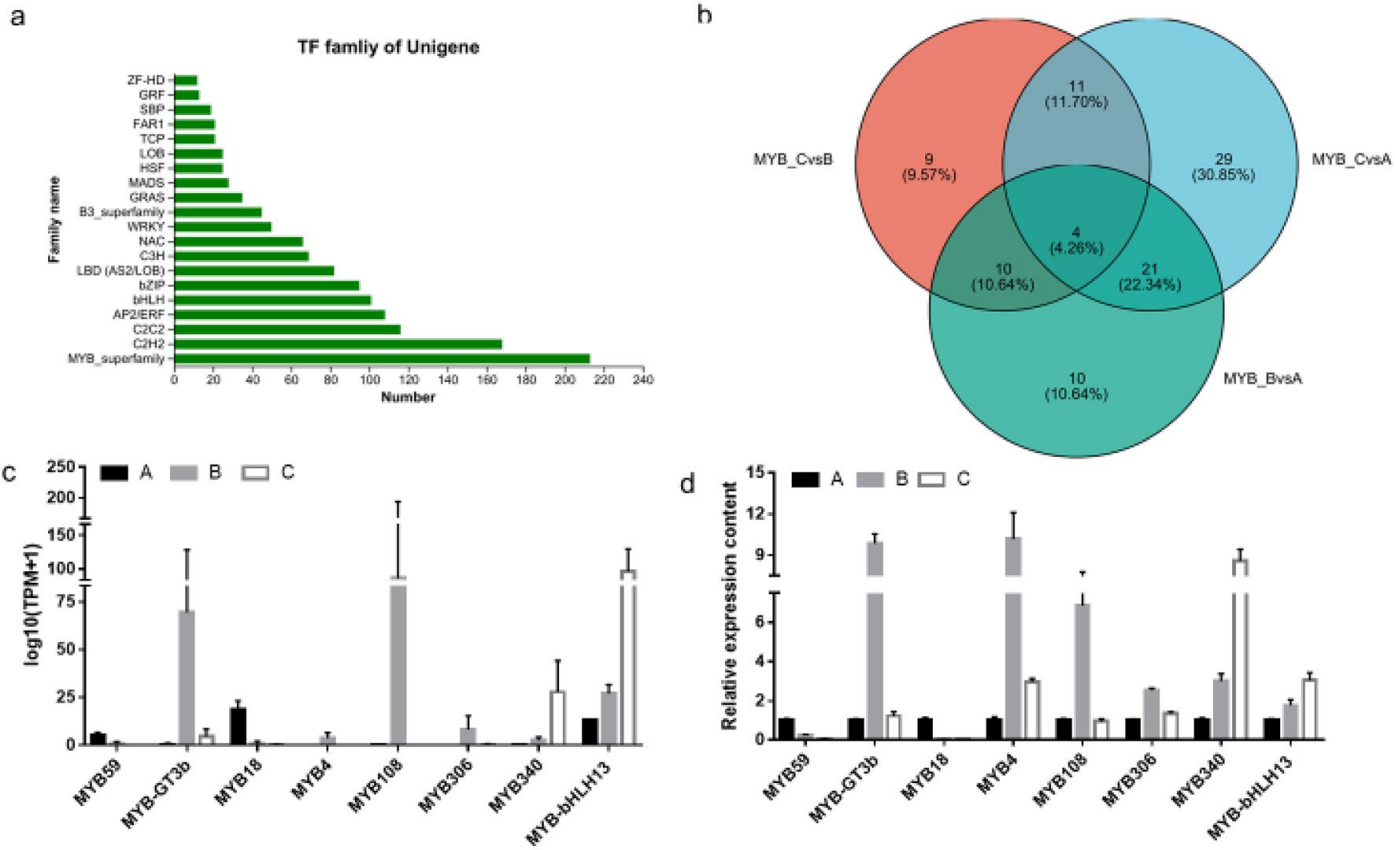
(**a**) Top 20 families of transcription factors. (**b**) Venn graph of gene sets, circles of different colors represent the unigene of a set of genes, and the value represents the number of unigene shared or unique among gene sets. (**c**) Expression analysis in this sequencing. (**d**) Relative expression of RT-qPCR.

TF is a type of protein that can combine with specific DNA sequences. It can recognize and bind to cis-acting elements in the upstream regulatory regions of genes through specific functional domains, which can activate or hinder gene expression. Transcription factor analysis was performed on the assembled gene/transcript to determine whether the gene/transcript was a transcription factor and its transcription factor family. According to the analysis of transcription factors, the top 20 families of transcription factors were the MYB superfamily, C2HA, and C2C2 (Fig. 7a). Among these, the number of nuigenes in the MYB superfamily was the highest, with 213 nuigenes. During the early development of Chieh-qua fruit, there were 94 unigenes of the MYB superfamily differentially expressed in three stages, among which there were 34 unigenes differentially expressed between B and C, 56 unigenes differentially expressed between C and A, and 45 genes differentially expressed between B and A. Four genes were compared in all three groups (Fig. 7b). Expression analysis of these differential myb superfamily unigenes is described in detail in the Supplementary Materials. RT-qPCR was performed to verify the gene expression. Among the eight selected unigenes of the MYB superfamily, the expression patterns of all unigenes in the three early stages of Chieh-qua fruit were consistent with those of the transcriptome sequencing (Fig. 7c,d). Myb59 and myb18 exhibited higher expression during fruit pollination for 1 week (Fig. 7c,d). myb4, myb-GT3b, myb108, and myb306 showed the highest expression levels in fruits that had been pollinated for 3 d (Fig. 7c,d). In addition, myb340 and myb-Bhlh13 showed higher expression levels during the unpollinated stage (Fig. 7c,d).

## Discussion

### Transcriptome differences of early fruit development after pollination of chieh-qua

Fruit expansion is influenced by the external environment and regulation of internal gene expression^25,26^. Transcriptome sequencing can promote the identification of whole gene expression and systematic regulatory mechanisms by analyzing fruits at different developmental stages at the transcriptional level. In this study, to screen the genes related to the early fruit development of chieh-qua, we sequenced and annotated the transcriptomes of three fruit development stages of the same Chieh-qua variety under the same conditions. The female flowers of Cucurbitaceae bear tender fruits when they bloom. After successful pollination, the fruit began to expand (Fig. 1a).At 7 dpp, fruit weight increased exponentially compared with that of unpollinated plants (Fig. 1b). Transcriptome sequencing of fruits at three different developmental stages produced 104,747 single genes. In the two bottle gourd varieties, only a small number of genes showed differences in fruit development at the same developmental stage^7^. In this study, there were three different early fruit development stages of the same variety of chieh-qua, and the gene expression in these three stages was significantly different (Table 3).

### Identification of DEGs in fruit transcriptome

To identify the corresponding genes related to early swelling and development of Chieh-qua fruit, we compared gene expression profiles of the transcriptome range among the libraries of the three developmental stages. When the transcription factor MYB was used as the gene set for analysis, the number of DEGs identified in the comparisons of A to C and A to B was significantly higher than that detected in the comparison of B to C (Fig. 7b). These changes were consistent with the morphological and physiological changes observed during the early development of Chieh-qua fruits after pollination, with little change between B and C and an exponential increase from B to A (Fig. 1a). However, in the whole-transcriptome analysis, the number of DEGs identified in the comparison of A to B and B to C was significantly higher than that detected in the comparison of A to C (Table 3). This result indicates that stage B is a key stage of development after pollination. The ratio of B to C is the difference in gene expression before and after pollination, and the ratio of B to A is the difference in the gene expression of fruit expansion after pollination.

### Functional annotation of transcriptome

A total of 56,807 unigenes (54.23%) were successfully annotated in at least one of the following databases: NR, Swiss-Prot, Pfam, GO, or KEGG. In the COG database, the number of nuigenes that may have the functions of ‘translation, ribosomal structure and biogenesis’ and ‘post translational modification, protein turn over, chaperones’ is in the top three. Unigenes with the largest number of comments in the GO database may function in metabolic processes, cellular processes, cells, cell parts, binding, and catalytic activity. In the KEGG database, the most frequently mentioned functions of unigenes were translation, folding, sorting and degradation, and carbohydrate metabolism.

### Transcription factors play a crucial regulatory role in plant development

Eight candidate genes homologous to the MYB family were obtained from the transcriptome data and compared with the genome data of winter melons in the NCBI database (Table S2). MYB340 and EBOII were homologous genes. EBOII was a transcription factor related to flowering in *Petunia hybrida*, and its transcript accumulation peaked during flowering^27^. Similarly, the expression of this gene in the Chieh-qua flowering period was significantly higher than in the two stages of fruit development after pollination (Fig. 7c,d). In *A. thaliana*, MYB59 is a transcription factor involved in plant growth and stress responses^28^. In chieh-qua, the transcript accumulation of the MYB59 homologous gene was the highest in the fruit of stage A (Fig. 7c,d). The EaMYB18 and SsMYB18 transgenic plants showed an effective tolerance to drought and cold stresses^29^. During Chieh-qua fruit development, the expression of the MYB18 homologous gene in the fruit at 7 dppwas significantly higher than that in the other two stages, which also indicated that the stronger stress resistance of old Chieh-qua fruits might be related to the regulation of transcription factor expression. In *A.thaliana*, Bhlh13 negatively regulates the JA signaling pathway^30^. During fruit expansion of chieh-qua, the expression of its homologous gene was negatively correlated with expansion. In Arabidopsis, Myb-GT3b is predominantly expressed in the foral buds and roots^31^. In this study, the homolog in Chieh-qua showed the highest expression at 3 dpp (Fig. 7c,d). However, the function of the MYB306-LIKE has not been reported. In this study, we found that the number of homologous genes was largestin fruit at 3 dpp. MYB108 and MYB4 are the transcription factors that regulate plant lignification^32^. In rice, MYB108 acts as a transcriptional repressor of lignin biosynthetic genes, and its mutation increases the lignin content in the cell walls^33^. In *A thaliana*, ARF17 regulates anther endothelial lignification by targeting MYB108 DNA^34^. In *A thaliana*, MYB4 is regulated by MKP-MAPK to inhibit leaf lignification^32^. There is also lignification between the flesh and peel during fruit development. After pollination, the homologous gene expression of MYB108 and MYB4 in the early stage of fruit development was the highest, which indicates that this stage is the key stage of fruit expansion and development and may be the node stage of fruit appearance and texture change.

## Conclusion

In the early development of Chieh-qua fruit, there are differences in gene expression at different stages before and after pollination and during fruit expansion, indicating that fruit expansion starts after pollination, and transcription factors play an important role in the expansion process.

These results indicate that MYB59, MYB-GT3b, MYB18, MYB4, MYB108, MYB306, MYB340, and MYB-bHLH13 play crucial roles in Chieh-qua fruit development, defense, and flowering. Overall, this study provides a basis for further investigation of MYB superfamily genes involved in early fruit expansion in chieh-qua.

### Supplementary Information

Supplementary Information.

Supplementary Table S1.

Supplementary Table S2.

## Data Availability

All data generated or analysed during this study are included in this published article [and its supplementary information files].
