# Supplementary material for: RNA-seq-based transcriptome profiling of early fruit development in Chieh-qua and analysis of related transcription factors
Source: Sci Rep. 2024 Jun 12;14:13489. doi: 10.1038/s41598-024-63871-6 (PMC11169226; doi:10.1038/s41598-024-63871-6)
Supplement: Supplementary file 1 — Supplementary Information. [file 41598_2024_63871_MOESM1_ESM.docx]

Supplementary Materials

Of the 213 myb_superfamily unigenes annotated in the transcriptome group, 94 members have obvious differences in expression levels in the early development of Chiqua fruit. The veen diagram in the main body has shown the difference in the number of these differential genes in two different periods (fig.7b), and the difference in the specific expression of these differential genes is expounded in the supplementary materials. Of the 34 differentially expressed genes between B and C, 9 were only different in B and C (fig.S1A), 10 were significantly different in B and C and also in B and A (fig.S2A), and 11 were significantly different in B and C and also in C and A (fig.S2B). There are 10 significant differences between B and A (fig.S1C), 29 significant differences between C and A (fig.S1B) and 21 unique unigenes(fig.S2C) in both groups. There are four unigene with obvious differences among the three groups (fig.S4), and their corresponding gene_ids are: TRINITY_DN32880_c1_g2, TRINITY_DN33604_c4_g10, TRINITY_DN35142_c2_g2, TRINITY_DN35924_c3_g2 (Table S1).


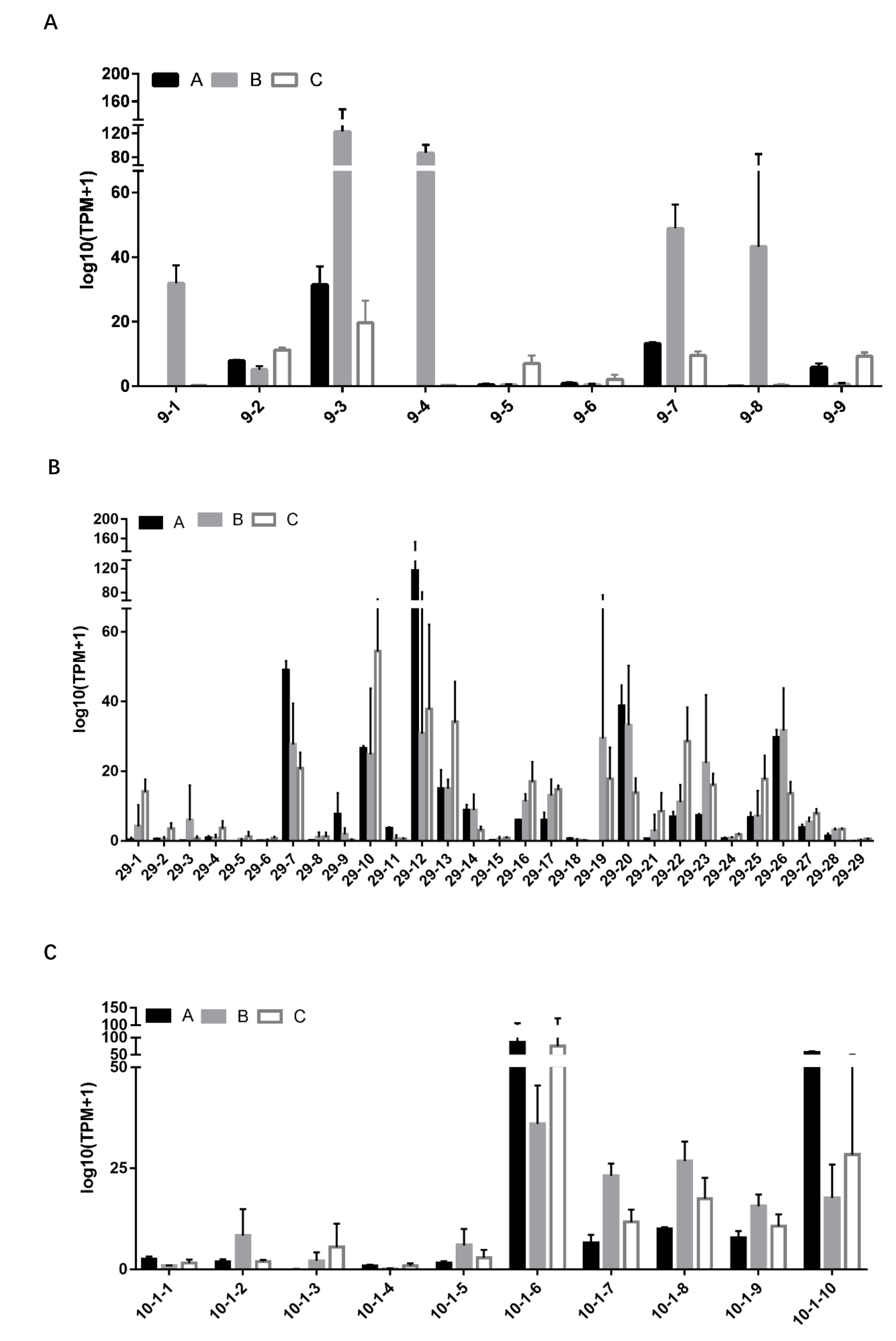


Fig S1. TPM values of myb_superfamily unigenes in 3 periods of chieh-qua fruits development. A: the TPM values have significant differences between in B and C; B: the TPM values have significant differences between in C and A; C: the TPM values have significant differences between in B and A.


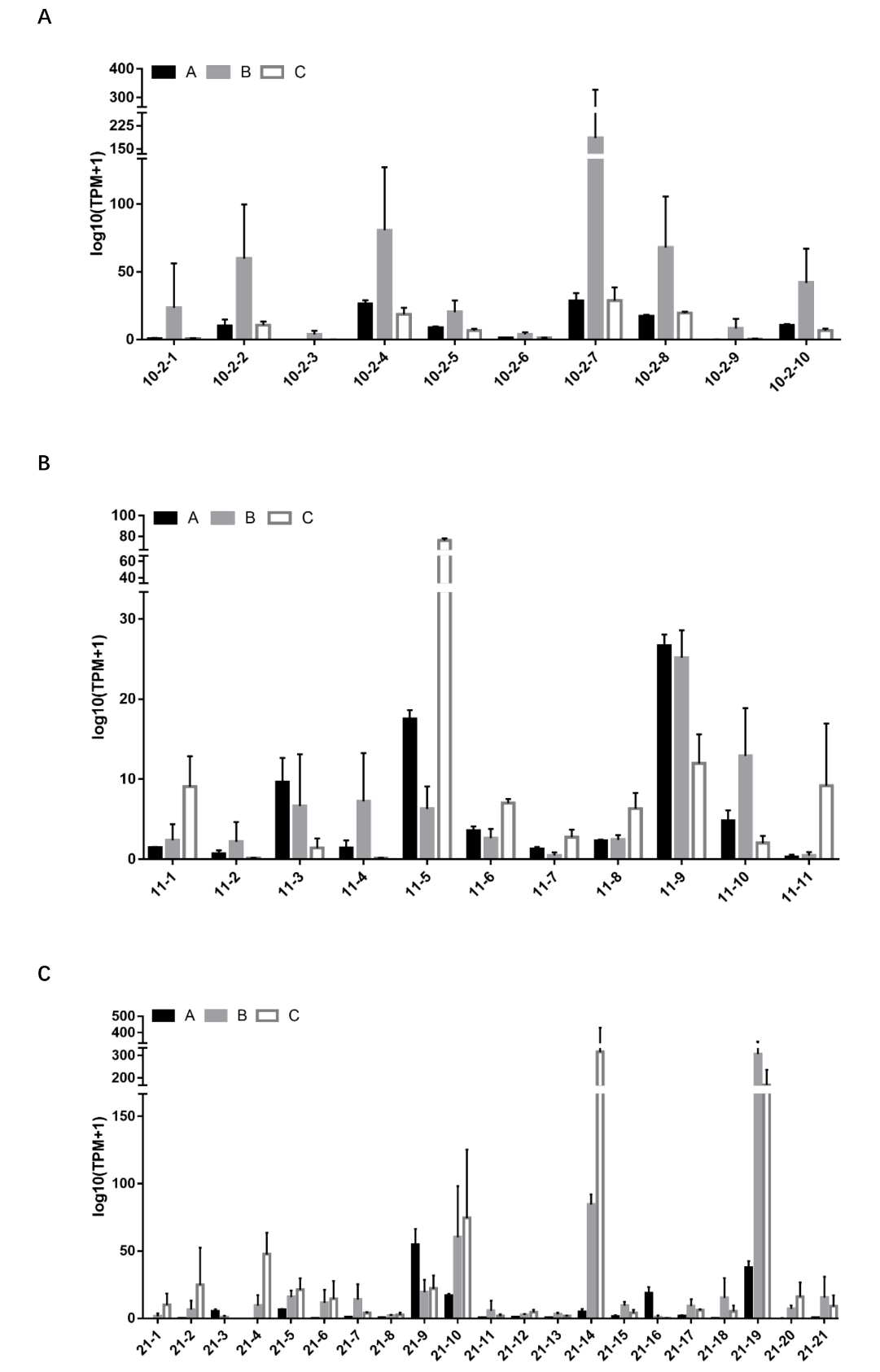


Fig S2. TPM values of myb_superfamily unigenes in 3 periods of chieh-qua fruits development. A: the TPM values have significant differences in C vs B and B vs A; B: the TPM values have significant differences in C vs B and C vs A; C: the TPM values have significant differences in C vs A and B vs A.


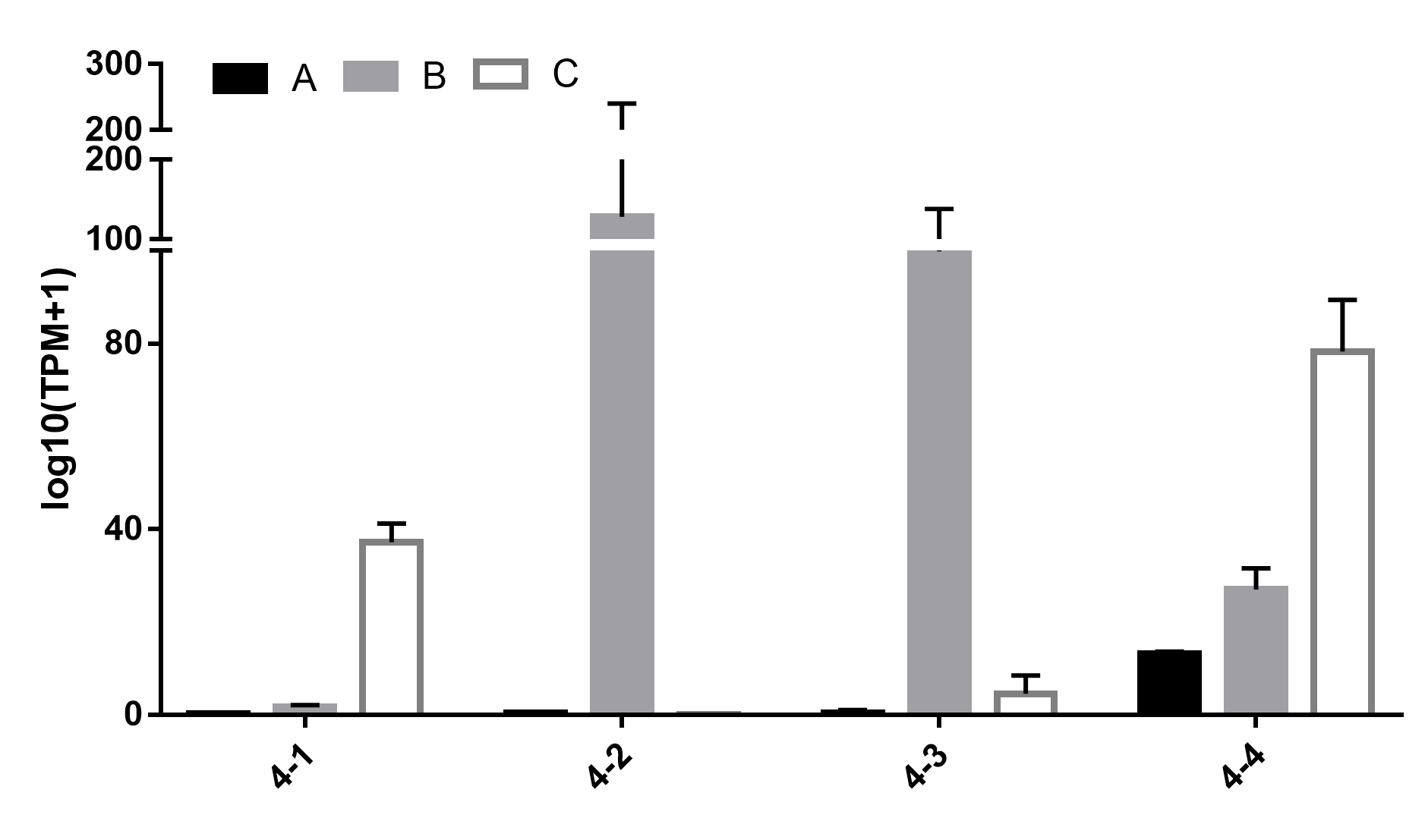


Fig S3. TPM values of myb_superfamily unigenes have significant differences in 3 periods of chieh-qua fruits development.
